# Fast Fluorine-18 labeling and preclinical evaluation of novel Mucin1 and its Folate hybrid peptide conjugate for targeting breast carcinoma

**DOI:** 10.1186/s41181-021-00127-y

**Published:** 2021-03-18

**Authors:** I. Al Jammaz, B. Al-Otaibi, Y. Al-Malki, A. Abousekhrah, S. M. Okarvi

**Affiliations:** Cyclotron and Radiopharmaceuticals Department, King Faisal Specialist Hospital and Research Centre, P.O. Box 3354, Riyadh, 11211 Kingdom of Saudi Arabia

**Keywords:** ^18^F-fluorination, ^18^F-fluoromucin 1, ^18^F-fluorofolate, Hybrid-peptide, Breast cancer

## Abstract

**Background:**

There is a need to develop new and more potent radiofluorinated peptide and their hybrid conjugates for multiple-receptors targeting properties that overexpress on many cancers.

**Methods:**

We have synthesized MUC1-[^18^F] SFB and MUC1-FA-[^18^F] SFB hybrid conjugates using a convenient and one-step nucleophilic displacement reaction. In vitro cell binding and in vivo evaluation in animals were performed to determine the potential of these radiolabeled compounds.

**Results:**

Radiochemical yields for MUC1-[^18^F] SFB and MUC1-FA-[^18^F] SFB conjugates were greater than 70% in less than 30 min synthesis time. Radiochemical purities were greater than 97% without HPLC purification, which makes these approaches amenable to automation. In vitro studies on MCF7 breast cancer cells showed that the significant amounts of the radiofluorinated conjugates were associated with cell fractions and held good affinity and specificity for MCF7 cells. In vivo characterization in Balb/c mice revealed rapid blood clearance with excretion predominantly by urinary as well as hepatobiliary systems for MUC1-[18F] SFB and MUC1-FA-[18F] SFB, respectively.

Biodistribution in SCID mice bearing MCF7 xenografts, demonstrated excellent tumor uptake (12% ID/g) and favorable kinetics for MUC1-FA-[^18^F] SFB over MUC1-[^18^F]SFB. The tumor uptake was blocked by the excess co-injection of cold peptides suggesting the receptor-mediated process.

**Conclusion:**

Initial PET/CT imaging of SCID mice with MCF7 xenografts, confirmed these observations. These results demonstrate that MUC1-FA-[^18^F] SFB may be a useful PET imaging probe for breast cancer detection and monitoring tumor response to the treatment.

## Introduction

Many tumor-associated antigens (TAAs) have been discovered and identified in the last decade and have provided new hope for the treatment of patients with malignant disease (Knutson et al., 2001; Brossart et al., 2000). The human epithelial mucin encoded by the gene MUC1 is an example of a tumor-specific antigen that is highly restricted on normal tissues but it is overexpressed on almost all human cancers (breast, ovarian, pancreatic, colorectal, lung, prostate, gastric cancers) and in particular, by primary and metastatic breast cancers (Kufe, 2009; Lakshminarayanan et al., 2012; Singh & Bandyopadhyay, 2007), thus making MUC1 a promising tumor-antigen with diagnostic as well as therapeutic potential in the management and treatment of cancer (Moore et al., 2004; Muller & Hanisch, 2002). Because overexpression of MUC1 correlates with high metastatic potential and poor patient survival, the ability to target such tumors may be highly beneficial in clinical settings (Kufe, 2009; Singh & Bandyopadhyay, 2007; Gendler, 2001). The measurement of circulating MUC1 levels in the serum, as determined by the CA15–3 assay (approved by the US Food and Drug Administration), has been used to monitor the clinical course of patients with breast cancer during treatment and to detect early disease recurrence; and the elevated levels of serum MUC1 are always linked with poor survival (Kufe, 2009; Singh & Bandyopadhyay, 2007). Important leads have suggested that MUC1 is a promising target for the development of vaccines and a number of MUC1 peptide based cancer vaccines are currently in clinical trials (Kufe, 2009; Lakshminarayanan et al., 2012; Singh & Bandyopadhyay, 2007). Development of small peptide-based agents for targeting MUC1 expressing tumors is more desirable because of their low immunogenic response and favorable biokinetics, together with high affinity and selectivity for target receptors. MUC1 is a breast cancer-associated transmembrane glycoprotein, of which the extracellular domain is formed by the repeating 20-amino acid sequence N-**PDTRP**APGSTAPPAHGVTSA-C (Luo et al., 2000; Brossart et al., 1999; Engelmann et al., 2001). The unique extracellular domain of MUC1 is defined by the presence of the amino acid sequence PDTRP, which is the minimal MUC1 core peptide sequence (shown in bold above) (Kufe, 2009; Moore et al., 2004; Pecher & Finn, 1996; Agrawal et al., 1998; Hussain et al., 1996; Grinstead et al., 2003). The same pentamer sequence is also recognized by several highly tumor-specific anti-mucin monoclonal antibodies (Pecher & Finn, 1996; Krambovitis et al., 1998; Girling et al., 1989; King et al., 1989; Xing et al., 1990). In addition, it has been suggested that the PDTRP core peptide sequence attains a structure closer to the native conformation and is believed to be immunodominant in humans (Singh & Bandyopadhyay, 2007; Grinstead et al., 2003; Krambovitis et al., 1998). Thus, it is anticipated that the high expression of MUC1 on breast cancer would allow target-specific imaging and therapy using synthetic MUC1-derived peptides. Peptide-based tumor receptor binding agents have attracted enormous attention as biological vehicles to deliver radioactivity to tumor cells for receptor-targeted imaging and radiotherapy. Several peptides are currently under investigation to determine their clinical potential as imaging and therapeutic agents for different cancers (McAfee & Neumann, 1996; Okarvi, 1999; Fishman et al., 1993; Boerman et al., 2000). Recently, the synthesis and in vitro and in vivo characterization of new ^99m^Tc-labeled-MUC1-derived peptide was reported suggesting the potential of this radiotracer for the targeted imaging of MUC1-positive breast cancer (Okarvi & Jammaz, 2009; Okarvi & Jammaz, 2016; Okarvi & Jammaz, 2019). However, more studies are required to determine the full potential of this peptide as a breast cancer imaging agent. On other hand, membrane-folic acid receptor is a glycosylphospstidylinositol protein that overexpressed in various epithelial cancers including breast cancer (Campbell et al., 1991; Antony, 1992). Meanwhile, this receptor is highly restricted in most normal tissues which make these tumors as excellent candidates for molecular targeting through the folate receptor system. Recent studies demonstrate that approximately 30% of breast cancers express folate receptor alpha (FRA) and suggest that as many as 70–80% of late-stage metastatic triple-negative breast cancer (TNBC) tumors express this receptor (Shannessy et al., 2012; Ginter et al., 2017). Overexpression of both the MUC1 and FRA receptors on the breast cancer highlight the potential application of the radiolabeled MUC1-conjugated folate hybrid peptide as dual-receptor-targeting imaging probes for breast carcinoma imaging. We hypothesized that the unique radiofluorinated MUC1-conjugated folic acid (FA) hybrid peptide targeting both the MUC1 and folate receptors would be superior in breast cancer targeting to the radiofluorinated MUC1 monomeric peptide or folate targeting only the folate or folate receptors. This may represent novel multiple-acting properties to the management and treatment for breast cancer disease with unmet medical needs. In this study, we have synthesized by solid-phase synthesis a novel MUC1-derived peptide based on PDTRP sequence and coupled it to a negatively-charged glutamic acid (Glu) residue as a spacer to keep the chelating-site distant from the receptor-binding region and to increase the hydrophilicity of the ^18^F-labeled peptide, which often resulted in faster renal excretion and improved target to background ratios. In addition, lysine (Lys) amino acid was terminally coupled with the former sequence to facilitate conjugation with FA and radiolabeling with fluorine-18 (^18^F). Finally, the Lys (GAMMA) amino group was coupled to the activated (GAMMA) FA residue to yield MUC1-FA hybrid peptide. Owing to the favorable nuclear and chemical characteristics of ^18^F for PET diagnostic imaging applications including an appropriate physical half-life (109.7 min) and low positron (β^+^) energy (0.64 MeV) (Okarvi, 2001; Varagnolo et al., 2000), we here present the radiolabeling with ^18^F and in vitro and in vivo evaluation of new MUC1 and its FA hybrid peptides for the diagnosis of breast cancer using PET imaging.

## Experimental

The chemicals used were all analytical reagent grades and used without further purification unless stated. Acetonitrile (ACN) and dimethylformamide (DMF) were kept over molecular sieves. High-Pressure Liquid Chromatography (HPLC) analysis was carried out on Econosil C-18 reversed-phase columns (semipreparative, 250 mm × 10 mm or analytical, 250 mm × 4.6 mm). The solvent system used for the semipreparative was non-linear gradient (eluant A, H_2_O with 0.1% trifluoroacetic acid (TFA); eluant B, ACN/H_2_O, 3/1 v/v with 0.1% TFA; gradient, 0 to 90% B, 90 to 90% B and 90 to 10% B over 10 min each at flow rate of 1.5 mL/min) and for the analytical was (eluant A, ACN with 0.1% TFA; eluant B, H_2_O with 0.1% TFA; gradient, 0 to 50% B, over 0–15 min and 50 to 0% B over 15-20 min at flow rate of 1.5 mL/min). A Jasco chromatographic system equipped with a variable wavelength ultraviolet monitor and in tandem with a Canberra flow through radioactivity detector was used. Ultraviolet absorption was monitored at 220 nm. Chromatograms were acquired and analyzed using BORWIN software. Mass spectroscopy was run on Quattra electrospray mass spectrometer (ES-MS).

### MUC1and MUC1-FA hybrid peptide conjugates

The MUC1 peptide analog was prepared utilizing the method reported previously (AlJammaz et al., 2014). Briefly, by solid-phase peptide synthesis (on a CS Bio peptide synthesizer, CA, USA) following standard Fmoc (9-fluorenylmethoxycarbonyl) chemistry, using Rink amide methylbenzhydrylamine (MBHA) resin on a 0.2 mmol scale. After incorporating all the desired amino acids, the N-terminal Fmoc-protecting group was removed and the peptide was cleaved from the resin followed by the removal of the other side-chain protecting groups using a mixture of TFA/H_2_O/dithiothreitol (DTT) 95:2.5:2.5 for 2 h at room temperature. The resin was removed by filtration, and the crude peptides were obtained by precipitation with cold diethyl ether (ether) followed by HPLC purification. For the synthesis of MUC1-FA hybrid peptide, the free epsilon (ɛ) amino group at terminal Lys residue on MUC1 peptide was coupled with FA via the activated gamma (γ) carboxyl moiety. The *N*-succinimidyl folate ester (folate-NHS, 10 μmol) dissolved in dimethylsulfoxide (DMSO, 100 μL) and followed by the addition of each peptide (10 μmol) and TEA (10 μmol). Reaction mixture stirred while shielded from light for 30 min at 50^o^ C. The MUC1-FA hybrid peptide was precipitated by addition of ACN (2 mL), centrifuged and then washed several times with ACN before drying. The identity and purity of the MUC1 peptide analog was characterized by mass spectrometry and HPLC.

### Fluorinated MUC1- and MUC1-FA-SFB hybrid peptide reference conjugates

**Scheme 1:**
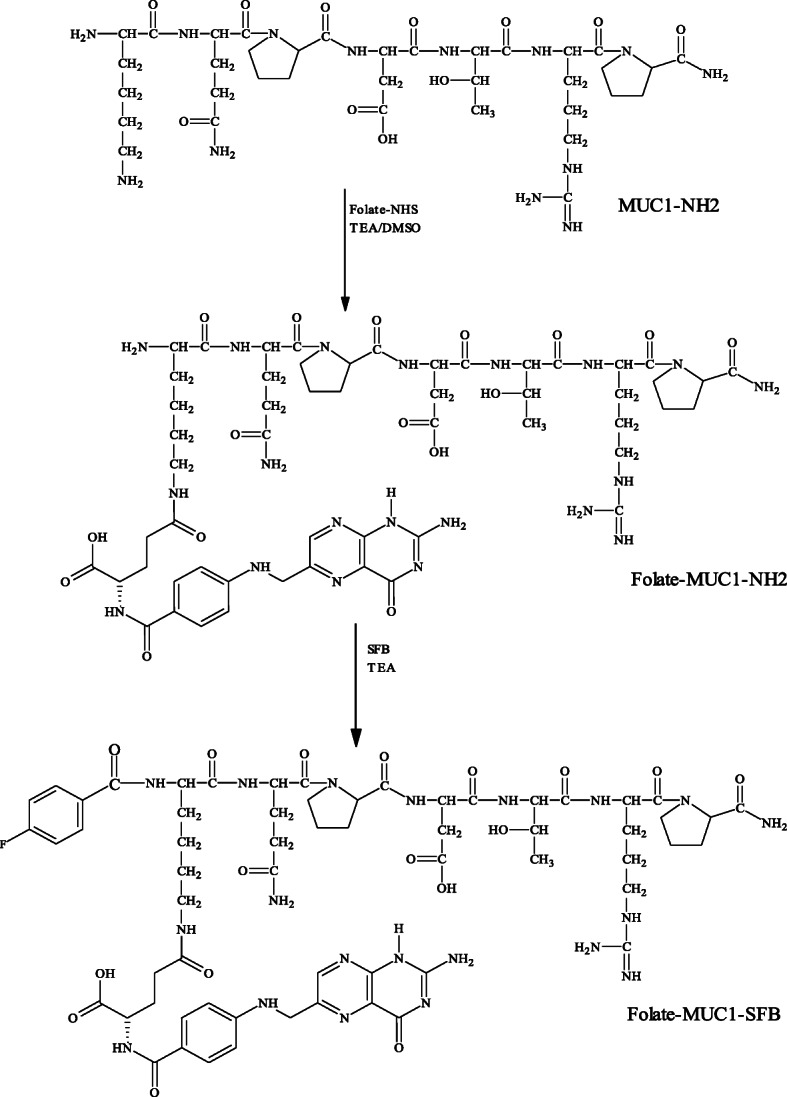
Synthesis of the MUC1-FA hybrid peptide and reference MUC1-FA-SFB hybrid peptide conjugate.

The reference fluorinated MUC1 and MUC1-FA peptide conjugates were prepared separately by coupling the precursors 4-fluorobenzoic acid to the non-receptor binding region through an amide linkage utilizing the methods reported previously (AlJammaz et al., 2014; AlJammaz et al., 2006) (Scheme 1). Briefly, MUC1 and MUC1-FA hybrid peptides (5 μmol each) were added to *N*-succinimidyl-4-fluorobenzoate (SFB, 8.5 μmol) in DMF (100 μL) and enough amount of triethylamine (TEA, 2 μL) to attain a pH 9. The reaction mixtures were heated for 20 min at 90^o^ C. This was followed by dilution with H_2_O (1 mL), loading onto Sep-Pak C-18 cartridge, washing with H_2_O (5 mL) and the peptide conjugate eluted with ethanol (EtOH, 1 mL). After solvents evaporation to dryness, white and slightly yellowish powders were separated and dried under vacuum to produce the reference MUC1-SFB or MUC1-FA-SFB hybrid peptide conjugates, respectively. The structures and purities of the fluorinated peptide analogs were characterized by mass spectrometry and HPLC.

### MUC1 and MUC1-FA 4-(N,N,N-trimethylammonium)benzoate.Triflate hybrid peptide precursors

**Scheme 2:**
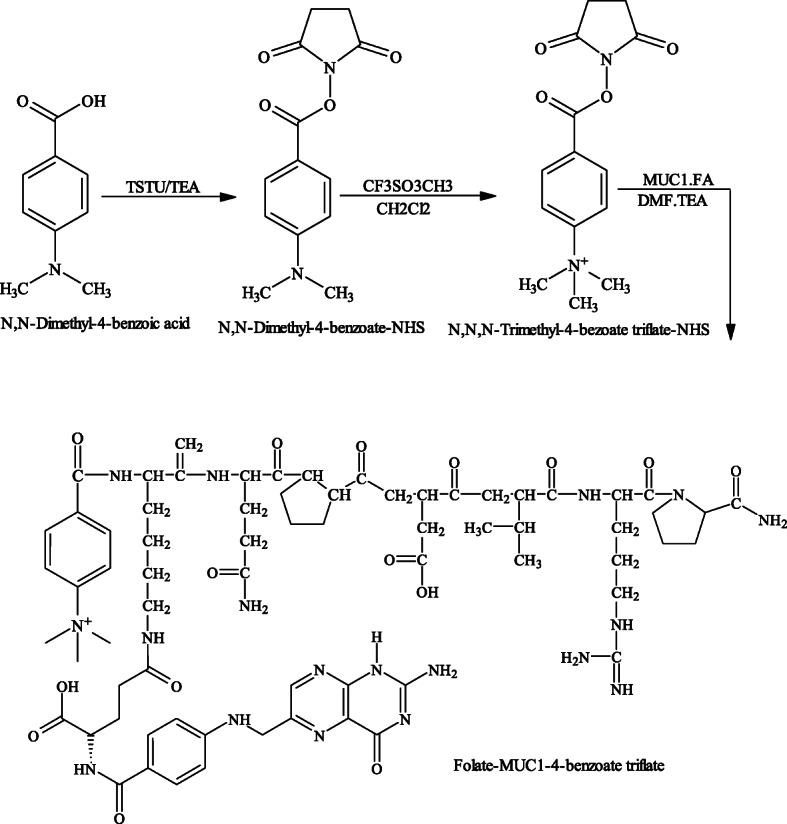
Synthesis of the MUC1-FA-4-benzoate hybrid peptide triflate precursor.

The MUC1 (3.0 mg, 3.5 μmol) and MUC1-FA hybrid peptide (3.0 mg, 2.37 μmol) conjugates were dissolved separately in DMF (100 μL) followed by the addition of TEA (1 μL, 6 μmol). *N*-succinimidyl 4-(*N,N,N*-trimethylammonium)benzoate.triflate (1.5 M equivalent) was then added and mixtures stirred at 80^o^ C for 15 min (Scheme 2). The MUC1- and MUC1-FA-triflate precursors were precipitated by addition of ACN (1 mL), centrifuged and then washed several times with ACN before drying under vacuum to yield white and slightly yellowish powders in 59% and 66%, respectively.

### Radiosynthesis of MUC1-[^18^F]- and MUC1-FA-[^18^F] SFB hybrid peptide conjugates

**Scheme 3:**
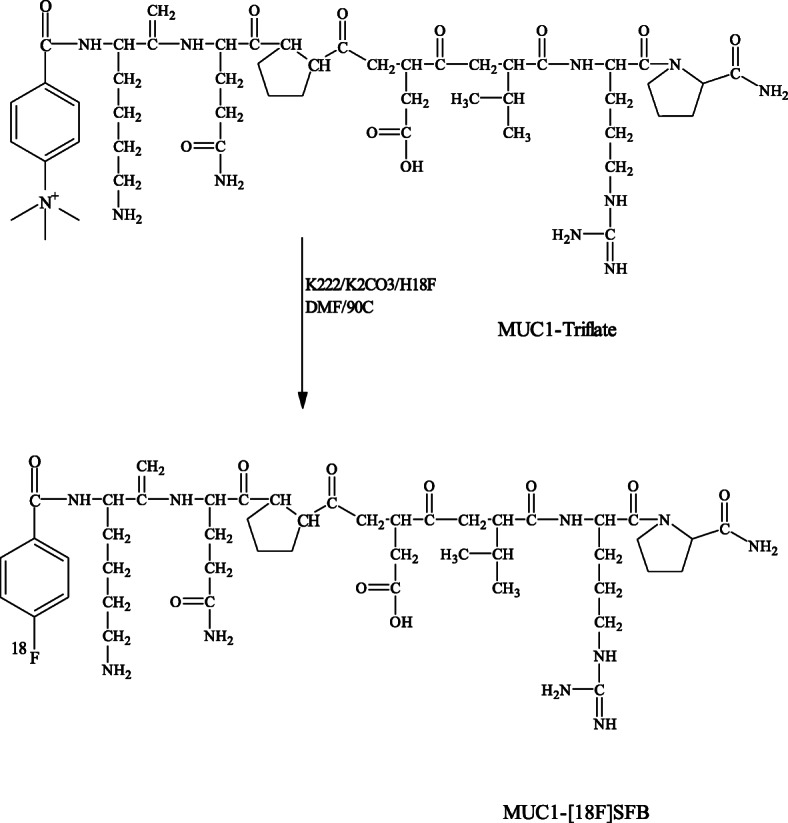
Radiosynthetic approach for MUC1-[^18^F] SFB peptide conjugate.

**Scheme 4:**
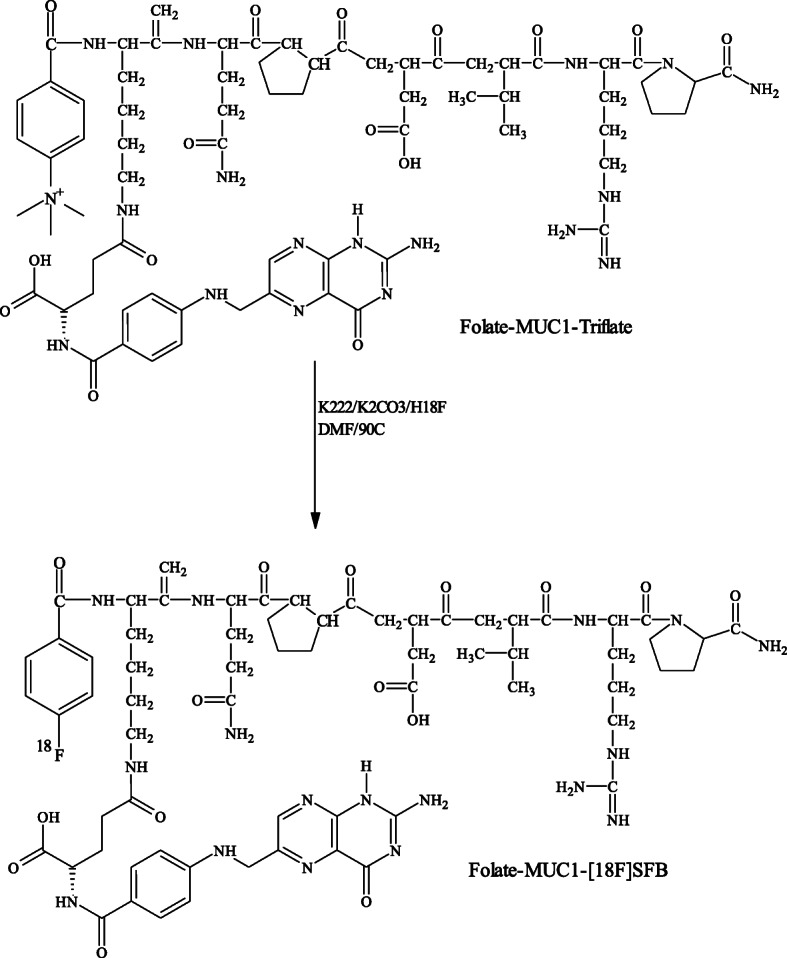
Radiosynthetic approach for MUC1-FA-[^18^F] SFB hybrid peptide conjugate.

Aqueous [^18^F]-fluoride was produced by the ^18^O(p,n)^18^F reaction. The fluoride activity (20-80 mCi, 740–2960 MBq) was trapped in Kryptofix 2.2.2 (5 mg) and potassium carbonate (1 mg) in ACN/H_2_O solution (950 μL/50 μL), dried by azeotropic distillation with aliquots of ACN. The solid residue was resolubilized in DMF (200 μL) and reacted in two different sealed vials containing the precursor Mucin 4-(*N,N,N*-trimethylammonium)benzoate.triflate peptide (50 μg, 50 nmol) and MUC1-FA 4-(*N,N,N*-trimethylammonium)benzoate.triflate hybrid peptide (50 μg, 35 nmol). The reaction mixtures were heated in capped 2 mL reaction-vials at 90^o^ C for 5 min, followed by the addition of H_2_O (1 mL) then passed through Sep-Pak C18 cartridge and washed with H_2_O (5 mL) to remove hydrophilic impurities (Schemes 3,4). Sep-Pak C18 cartridge was then dried with a steady stream of nitrogen, MUC1-[^18^F] SFB and MUC1-FA-[^18^F] SFB hybrid peptide conjugates were eluted with EtOH (1 mL). EtOH solutions were dried and residues were then re-solubilized in saline (0.9% NaCl, 1 mL each) before passing through 0.22 μm pore membrane filter for further studies.

### Partition coefficient

100 μL of MUC1-[^18^F] SFB and MUC1-FA-[^18^F] SFB hybrid peptide conjugates were added into test tubes containing 1 mL of each n-octanol and buffered H_2_O (pH = 7.3). The tubes were shaken for 1 min. After partial separation of the phases by gravity, 0.7 mL of each phase was transferred to separate tubes and centrifuged at 5000 rpm for 5 min. Duplicate 0.2 mL aliquots of each phase were taken for radioactivity measurement and the partition coefficient was determined by the function: Partition coefficient = Log_10_ (counts in n-octanol layer/counts in aqueous layer).

### Stability in plasma

For stability in plasma, the purified MUC1-[^18^F] SFB and MUC1-FA-[^18^F] SFB hybrid peptide conjugates (50 μL, 20 μCi each) were incubated with human plasma (500 μL) in duplicate at 37^o^ C for 2 h. This was followed by precipitation using a mixture of ACN/EtOH (400 μL, 1:1 v/v) and centrifugation at 5000 rpm for 5 min. The supernatant layer was then analyzed by HPLC under the conditions described above. The experiments were repeated 3 times.

### In vitro cell binding

The cell-binding activity of the MUC1-[^18^F] SFB and MUC1-FA-[^18^F] SFB hybrid peptide conjugates were measured on the human MCF7 breast cancer cell line (ATCC, Rockville, MD). MCF7 cell line was grown in RPMI-1640 culture media with 10% fetal bovine serum (FBS) in tissue culture flasks. 24 h prior to conducting the cell-binding assay, media was replaced with RPMI-1640 without further addition of FBS. Confluent cultures were harvested by trypsinization, and 6 × 10^6^ cells were suspended in 1.8 mL of sterile saline for binding assay. Approximately 300,000 cells (in 0.3 mL of sterile saline) were incubated with various amounts of the purified MUC1-[^18^F] SFB and MUC1-FA-[^18^F] SFB hybrid peptide conjugates ranging from 0.3–18 nM in duplicate for 60 min at room temperature. Incubation was terminated by dilution with cold saline (0.3 mL) and cells were pelleted by centrifugation. The cell-pellets were then washed with cold saline to remove unbound radioactivity and centrifuged to collect supernatants. Radioactivity in the cell-pellets (total bound) and washings (unbound) were measured in a γ-well counter. Non-specific binding was determined in the presence of approximately 100-fold excess of each unlabeled MUC1 and unlabeled MUC1-FA- hybrid peptide. Specific binding was calculated by subtracting the non-specific bound radioactivity from that of the total binding. The data were analyzed by a non-linear regression analysis program (Graph-Pad Software Inc., San Diego, CA, USA). All binding data were corrected for non-specific binding and presented as the mean ± S.D. The experiments were repeated 3 times.

### In vivo biodistribution

Approval for the animal protocol used in this study was obtained from the Institutional Animal Care and Use Committee. Animal biodistribution experiments were performed according to the international regulations governing the safe and humane use of laboratory animals in research (Guide for the Care of and Use of Laboratory Animals, 1996). The biodistribution was performed in normal female Balb/c mice (*n* = 4 at each time point; body mass 20-25 g) to ascertain the in vivo distribution profile of the MUC1-[^18^F] SFB and MUC1-FA-[^18^F] SFB hybrid peptide conjugates. Mice were injected via the lateral tail vein with 100 μL of the radiotracers formulated in saline. Each dose contained ~ 10 μg of the peptide and ~ 20 μCi (740 kBq) of radioactivity. Animals were sacrificed at different time intervals and tissues of interest were dissected, weighed and assayed for radioactivity. The percentage of the injected dose per gram (% ID/g) was then calculated by counting all tissues in a γ-well counter using a stored sample of the injection solution as a standard to estimate the total dose injected per mouse.

### In vivo tumor targeting

Human MCF7 xenografts SCID female mouse models were used for in vivo tumor targeting experiments. For the implantation of tumor xenografts, approximately 3 × 10^6^ MCF7 cells in suspension of 100 μL sterile saline were injected subcutaneously into the right thigh of each mouse. Tumors were allowed to grow for 4-6 weeks by which tumors had reached weights of ~ 500 mg. Animals were injected with 20 μCi (740 kBq) of the radiotracers. For the blocking studies, each animal was intravenously injected with excess cold of MUC1 and MUC1-FA hybrid peptide (~ 100 μg) 30 min prior to the radiotracers injection. The animals (*n* = 4 per group) were sacrificed at 60 min post radiotracers injection (p.i.) and the % ID/g for the tumor and major organs was calculated as described above.

### In vivo Nano PET/CT imaging

PET/CT scans were performed using a preclinical NanoPET/CT scanner (Mediso, Hungary) on MCF7 tumor-bearing SCID female mice (8 weeks old). MUC1-[^18^F] SFB and MUC1-FA-[^18^F] SFB hybrid peptide conjugates (7.4 MBq/100 μL) were injected into each mouse (*n =* 2-3) through tail vein and placed in the Nano PET/CT scanner with continuous O_2_ and 2% isoflurane supply. 60 min post tail vein injection of the radiotracers, the mouse was imaged for 30 min PET/CT acquisition time. A static scan was acquired at 60 min post-injection. CT scan was performed using the following parameters: X-ray voltage = 50 kVp, Exposure time = 300 ms. A total projection of 288 projects over 360° of rotation were acquired and reconstructed using a cosine filter. This was followed by a PET data acquisition with the following parameters: 5-ns coincidence window and 400–600 keV energy window in 1–5 coincidence mode. Crystal efficiency correction was also applied, with a ring difference of 8, and the images were reconstructed by a three-dimensional ordered-subsets; exception maximum algorithm (subsets, 4; iterations, 6). Pixel size was 0.3 mm. The acquired data in these studies were analyzed by InterVeiw FUSION software developed by Mediso. Two to three animals were imaged at the specified time point.

### Statistical analysis

Data are expressed as mean ± S.D. where appropriate. For data comparisons, a Student’s *t* test was performed of the mean values using Graph-Pad Software (Graph-Pad Software Inc., San Diego, CA, USA). A probability value of *P* < 0.05 was considered statistically significant.

## Results and discussion

### Organic chemistry

The MUC1 peptide investigated was successfully prepared in good yields (30%) by solid-phase synthesis according to standard Fmoc/HBTU methodology. After completion of solid-phase synthesis, free ɛ amino group on lysine terminus of MUC1 peptide was coupled to activated γ-folate carboxylate to furnish MUC1-FA hybrid peptide in 60–70% yield. The purities of these peptides were found to be greater than 95% as characterized by analytical HPLC and the calculated molecular masses for MUC1 and MUC1-FA hybrid peptides were in agreement with the experimentally found ES-MS [M + 1]^+^ values 841 and 1264, respectively. The synthesis of amide-linked reference MUC1-SFB and MUC-FA-SFB hybrid peptide conjugates entailed several sequences of reactions as illustrated in Scheme 1. All these conjugates were obtained as white and slightly yellowish powders, respectively, and the overall yields and chemical purities were greater than 70%. The calculated molecular mass for the fluorinated MUC1-SFB and MUC1-FA-SFB hybrid peptide conjugates were 962.1 and 1378.5 and were in agreement with the found molecular ion ES-MS [M + 1]^+^ = 963 and 1379, respectively. The key precursors 4-*N,N,N-*trimethylammonium benzoate-MUC1 and MUC1-FA-triflate precursors were prepared by reacting *N*-succinimidyl 4-N,N,N-trimethylammonium benzoate with MUC1 and MUC1-FA hybrid peptides to furnish both peptide triflate precursors up to 70% yield and > 95% chemical purity (Scheme 2). The calculated molecular mass for MUC1 and MUC1-FA hybrid peptide triflate conjugates were 1003.9 and 1418.6, respectively and were in agreement with the attained ES-MS [M + 1]^+^ = 1004 and 1419, respectively.

### Radiochemistry

**Fig. 1:**
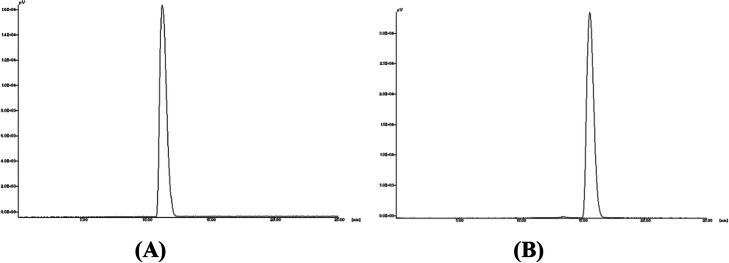
HPLC chromatograms of MUC1-[^18^F] SFB peptide (A) and MUC1-FA-[^18^F] SFB hybrid peptide conjugates (B)

The synthetic approaches for the preparation of MUC1-[^18^F] SFB and MUC1-FA-[^18^F] SFB hybrid peptide conjugates entailed only a one-step reaction (Schemes 3, 4). Both precursors were treated using catalyzed nucleophilic no-carrier-added radiofluoride produced by the ^18^O(p,n)^18^F nuclear reaction on ^18^O-enriched (98%) water and Kryptofix 222 as nucleophilic catalyst in anhydrous DMF at 80^o^ C for 5 min. The radiofluorinated peptides were purified by C-18 Sep-Pak column and the overall radiochemical yields for MUC1-[^18^F] SFB and MUC1-FA-[^18^F] SFB hybrid peptide conjugates were up to 70% (based on starting [^18^F]-fluoride), in less than 30 min total synthesis time. As shown in Fig. 1, radiochemical purities of MUC1-[^18^F] SFB and MUC1-FA-[^18^F] SFB hybrid peptide conjugates were always greater than 95%, as determined by HPLC, with retention times of 11.4 and 15.4 min, respectively. This synthetic approach holds considerable promise as a rapid and efficient method amenable for automation for the radiofluorination of peptides, with high radiochemical yield and short synthesis time. In addition, the calculated partition coefficient for MUC1-[^18^F] SFB and MUC1-FA-[^18^F] SFB hybrid peptide conjugates were found − 2.030 ± 0.09 and − 1.14 ± 0.08, respectively, representing a low lipophilic characteristic for both conjugates. Moreover, the specific activities for MUC1-[^18^F] SFB and MUC1-FA-[^18^F] SFB hybrid peptide conjugates were always greater than 1000 mCi/μmol. Hence, these radiofluorinated-peptide conjugates could be suitable for biochemical studies, such as radioligand binding assays.

### Stability in plasma

The proteolytic degradation of the MUC1-[^18^F] SFB and MUC1-FA-[^18^F] SFB hybrid peptide conjugates were determined in human plasma in vitro. HPLC analysis of the plasma revealed that both radioconjugates remained sufficiently stable (90.0 ± 10.0%) during incubation at 37^o^ C for at least 2 h, demonstrating a high in vitro stability of these radioconjugates.

### In vitro cell binding

**Fig. 2:**
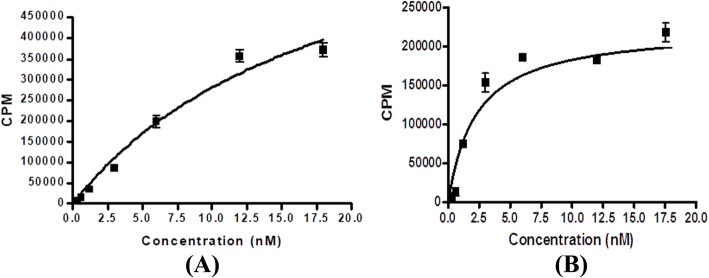
Determination of binding affinity (*K*_d_) values (*n =* 3) of MUC1-[^18^F] SFB (A) and MUC1-FA-[^18^F] SFB hybrid peptide conjugates in MCF7 breast cancer cell line

The binding affinities (*K*_d_) for MUC1-[^18^F] SFB and MUC1-FA-[^18^F] SFB hybrid peptide conjugates were evaluated by using the MCF7 breast cancer cell line. The *K*_d_ values of these bioconjugates were determined by saturation assays (Fig. 2). The results demonstrate that the conjugate MUC1-FA-[^18^F] SFB hybrid peptide has higher binding affinities to MCF7 breast cancer cell line than MUC1-[^18^F] SFB conjugate (4.06 ± 0.15 and 19.31 ± 4.23 nM). This result indicates that the binding affinity for the new MUC1-[^18^F] SFB peptide conjugate is comparable to that of ^99m^Tc-MAG_3_-MUC1 (Okarvi & Jammaz, 2009; Okarvi & Jammaz, 2016). It is generally expected that hybrid molecules, capable of targeting dual receptor systems, may improve the tumor-targeting efficacy of the compound by increasing the accumulation of radioactivity in the tumors. This is because more tumor cells would be targeted with hybrid radioligand than would be possible with only a single radioligand. The same trend was true for MUC1-FA-[^18^F] SFB hybrid peptide conjugate, where the affinity increased by five folds after the conjugation of MUC1 to folic acid. Additional studies are required to get a better insight into this binding variation.

### In vivo biodistribution and tumor uptake

The biodistribution data in normal female Balb/c mice for MUC1-[^18^F] SFB and MUC1-FA-[^18^F] SFB hybrid peptide conjugates at 10, 60, and 120 min p.i. are shown in Table 1. The results of biodistribution for MUC1-[^18^F] SFB peptide conjugate generally demonstrate fast and efficient clearance from the blood and most of the organs and tissues. Peptide conjugate MUC1-[^18^F] SFB, displayed moderate radioactivity uptake in the kidneys suggesting that the major route of elimination was the urinary system. Whereas, MUC1-FA-[^18^F] SFB hybrid peptide conjugate revealed fast clearance from the blood and slightly higher radioactivity accumulations in most of the organs. However, significant radioactivity accumulation in the kidneys and intestines was observed suggesting that the route of elimination was the both urinary and hepatobiliary systems. The higher radioactivity uptake in kidneys for MUC1-FA-[^18^F] SFB hybrid peptide in comparison with MUC1-[^18^F] SFB peptide is likely a result of the presence of folate receptor in the proximal tubules. These results are in agreement with the partition coefficient measurements performed previously. In addition, the lower bone activity for these conjugates can be due to the high stability of the carbon-fluorine bond.

**Table 1:** Biodistribution of the MUC1[^18^F] SFB and MUC1-FA-[^18^F] SFB hybrid peptide conjugate in normal mice

	MUC1[^**18**^F]SFB			MUC1-FA-[^**18**^F]SFB		
	10 min	60 min	120 min	10 min	60 min	120 min
**Blood**	9.77 ± 1.02	2.80 ± 0.50	1.34 ± 0.24	3.63 ± 0.24	0.97 ± 0.14	0.39 ± 0.14
**Liver**	12.10 ± 0.39	2.88 ± 0.66	1.59 ± 0.57	7.17 ± 1.75	2.61 ± 0.69	1.0 ± 0.28
**Lung**	4.52 ± 0.55	1.34 ± 0.16	0.66 ± 0.12	7.84 ± 1.74	4.19 ± 0.35	1.15 ± 0.51
**Kidney**	11.42 ± 2.41	3.43 ± 0.45	1.09 ± 0.45	11.09 ± 1.41	5.36 ± 0.50	2.39 ± 0.45
**Intestine**	3.48 ± 1.01	1.94 ± 0.23	0.65 ± 0.16	4.35 ± 0.91	4.11 ± 0.34	3.29 ± 0.55
**Heart**	4.77 ± 0.69	1.77 ± 0.24	0.44 ± 0.23	3.57 ± 0.19	1.17 ± 0.24	0.41 ± 0.12
**Muscle**	1.29 ± 0.09	1.13 ± 0.46	0.69 ± 0.17	2.05 ± 0.45	0.58 ± 0.24	0.15 ± 0.04
**Bone**	1.34 ± 0.16	1.31 ± 0.28	0.75 ± 0.27	0.20 ± 0.09	0.02 ± 0.01	0.01 ± 0.00
**Spleen**	2.77 ± 0.25	1.06 ± 0.19	0.24 ± 0.09	2.72 ± 0.65	2.99 ± 0.89	1.96 ± 0.82

The values are average of % injected dose/gram ± SD for *n =* 4

**Fig. 3:**
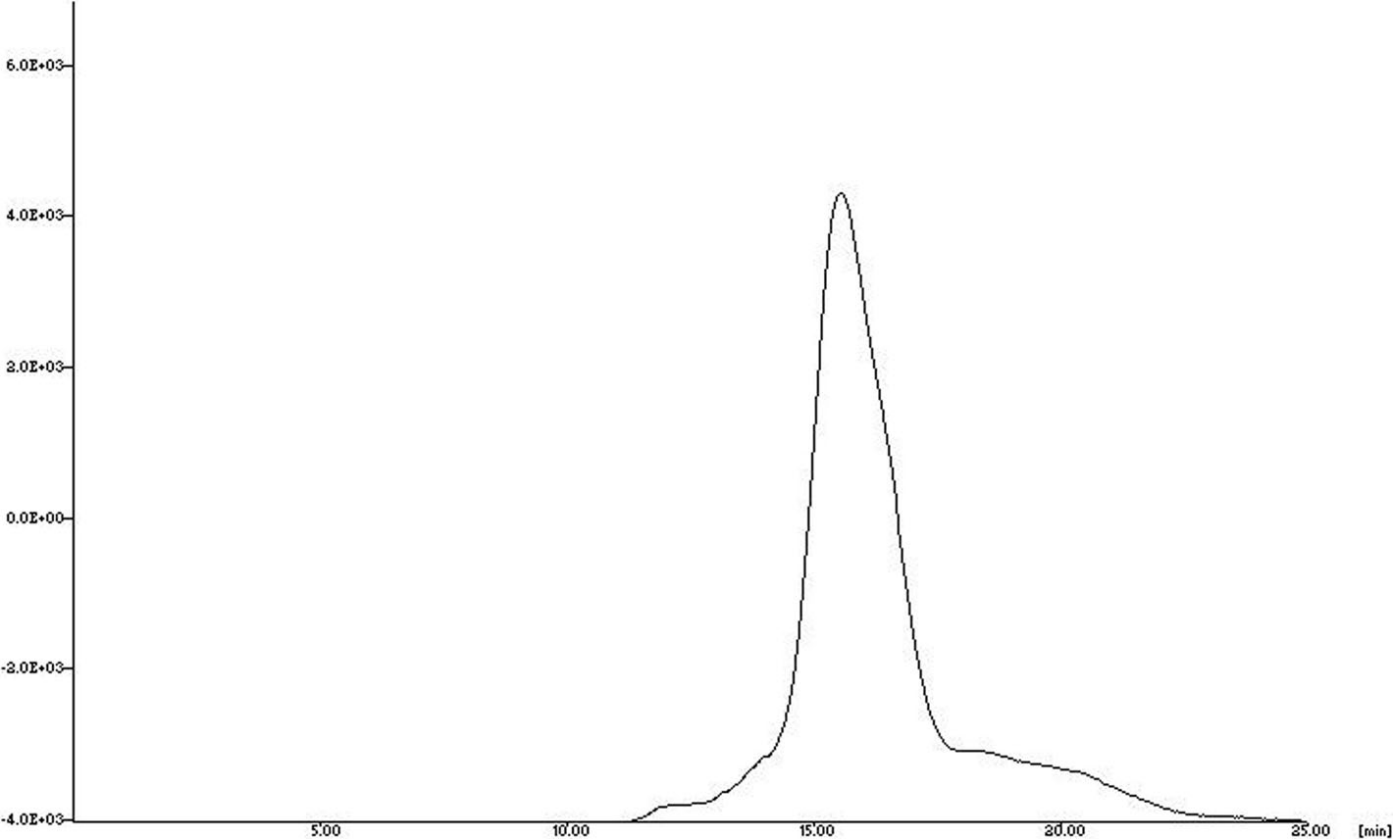
Radio-HPLC analysis of the urine sample of MUC1-FA-[^18^F]SFB obtained from the mouse during biodistribution at 60 min post-injection

In female SCID mice bearing human MCF7 cell line xenografts, MUC1-[^18^F] SFB peptide conjugate displayed a rapid clearance from the blood, with 0.35 ± 0.11% ID/g of radioactivity remained in the blood after 60 min p.i. (Table 2). When compared with normal mice biodistribution studies, a moderate uptake of MUC1-[^18^F] SFB peptide conjugate was found in most organs and tissues. The exact reason for the low uptake is unclear, but we assume that this behavior may be attributed to the nature of mouse strain. Good tumor uptake was observed for MUC1-[^18^F] SFB peptide conjugate (2.93 ± 0.12% ID/g) at 60 min p.i. and tumor-to-blood and tumor-to-muscle ratios obtained were 8.37 and 13.32, respectively. A fairly high uptake by the tumors combined with good tumor to background uptake ratios advocating the possible potential of this tumor-specific antigen peptide for targeting human breast cancer. In a blocking study where 100 μg of MUC1 peptide was administered 30 min before the injection of MUC1-[^18^F]-SFB peptide conjugate reduced the uptake in the tumors by approximately 45% (1.61 ± 0.22% ID/g blocked vs. 2.93 ± 0.12% ID/g unblocked, *P* = 0.01), highlighting the specificity of the MUC1-[^18^F] SFB peptide for respective MUC1-positive breast cancer cell line. No marked influence of the blocking dose was observed in other major organs and tissues. It is worth mentioning that the biodistribution and tumor uptake profiles of MUC1-[^18^F] SFB peptide conjugate is superior to the ^99m^Tc-MAG_3_-MUC1 peptide reported previously (Okarvi & Jammaz, 2009; Okarvi & Jammaz, 2016; Okarvi & Jammaz, 2019). Similarly, the hybrid MUC 1-FA-[^18^F] SFB peptide conjugate displayed fast clearance from the blood and an excellent tumor uptake of 12.03 ± 0.57% ID/g at 1 h p.i. The uptake value in the tumor was always higher than the radioactivity in other organs and much higher than uptake values for the blood and muscle. The tumor to blood and tumor to muscle ratios were 17.9 and 19.8, respectively. The high uptake by the tumors combined with good tumor to background uptake ratios indicating the potential of this peptide for targeting human breast cancer. The high tumor uptake was dramatically decreased (12.03 ± 0.57% vs. 1.37 ± 0.22% ID/g, *P* = 0.02) in the presence of a blocking dose of the full sequence MUC1-FA. This indicates the specificity of the MUC1-FA-[^18^F] SFB hybrid peptide for MUC1-positive breast cancer cell line. A marked influence of the blocking dose was observed also in kidneys which is a result of the masking of folate receptor in the proximal tubules. The rapid clearance from the blood and the excretion mainly by renal pathway for MUC1-[^18^F] SFB peptide and by renal as well as hepatobiliary pathways for MUC1-FA-[^18^F] SFB hybrid peptide is attributed to the hydrophilic characteristic of former in comparison with the latter as demonstrated in the partition coefficient measurement. The amounts of radioactivity of all the conjugates excreted into the urine at the time of sacrifice (60 min p.i.) were collected and examined by HPLC to determine the in vivo stability MUC1-[^18^F] SFB and MUC1-FA-[^18^F] SFB hybrid peptide conjugates. Radio-HPLC analysis of the urine sample showed that a significant amount of the radioactivity (> 90%) was still associated with the radioconjugates (Fig. 3). These findings demonstrate that these radioflorinated conjugates are not prone to rapid in vivo degradation and correlate well with the findings of high metabolic stability in human plasma in vitro.

**Table 2:** Biodistribution of the MUC1-[^18^F] SFB and MUC1-FA-[^18^F] SFB hybrid peptide conjugates in tumor-bearing SCID mice

	MUC1-[^**18**^F]SFB		MUC1-FA-[^**18**^F]SFB	
	60 min	60 min blocked	60 min	60 min blocked
**Blood**	0.35 ± 0.11	0.39 ± 0.15	0.67 ± 0.07	0.52 ± 0.05
**Liver**	0.85 ± 0.07	0.55 ± 0.17	3.20 ± 0.36	2.14 ± 0.13
**Lung**	1.39 ± 0.42	0.99 ± 0.22	2.96 ± 0.40	2.05 ± 0.49
**Kidney**	1.28 ± 0.03	1.20 ± 0.23	5.14 ± 0.53	1.86 ± 0.61
**Intestine**	0.21 ± 0.01	0.19 ± 0.08	4.36 ± 0.77	2.50 ± 0.65
**Heart**	0.38 ± 0.03	0.31 ± 0.07	1.98 ± 0.11	1.71 ± 0.12
**Muscle**	0.22 ± 0.13	0.24 ± 0.09	0.61 ± 0.05	0.25 ± 00.09
**Bone**	0.11 ± 0.01	0.15 ± 0.03	0.70 ± 0.14	0.30 ± 0.21
**Spleen**	1.07 ± 0.26	0.88 ± 0.11	2.95 ± 0.37	2.28 ± 0.71
**Tumor**	2.93 ± 0.12	1.61 ± 0.22	12.03 ± 0.57	1.37 ± 0.22

The values are average of % injected dose/gram ± SD for *n =* 4

### In vivo Nano PET/CT imaging

**Fig. 4:**
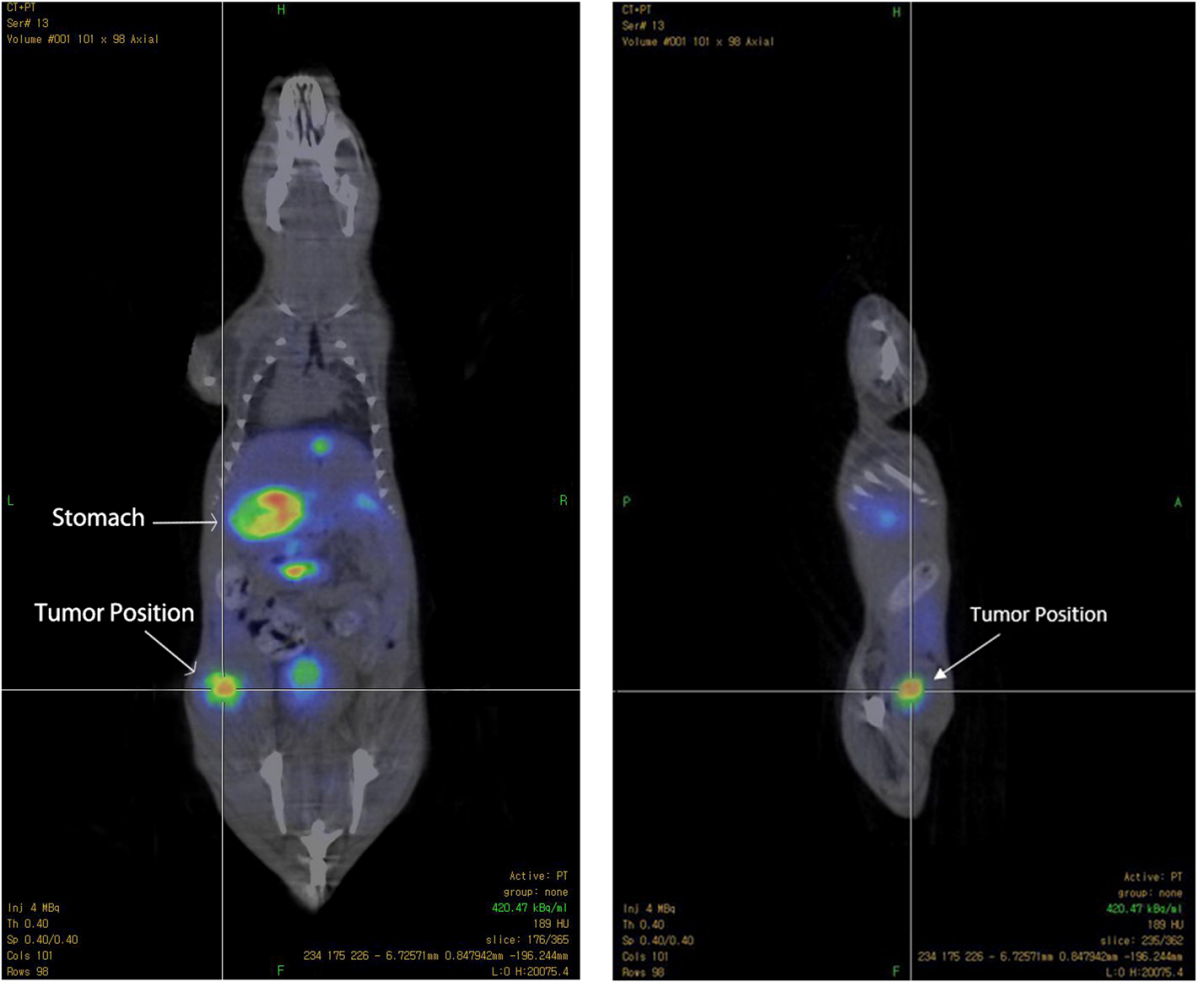
Coronal and sagittal images of tumor-bearing mouse after 1 h post-injection using 5 MBq MUC1-[^18^F] SFB peptide conjugate

**Fig. 5:**
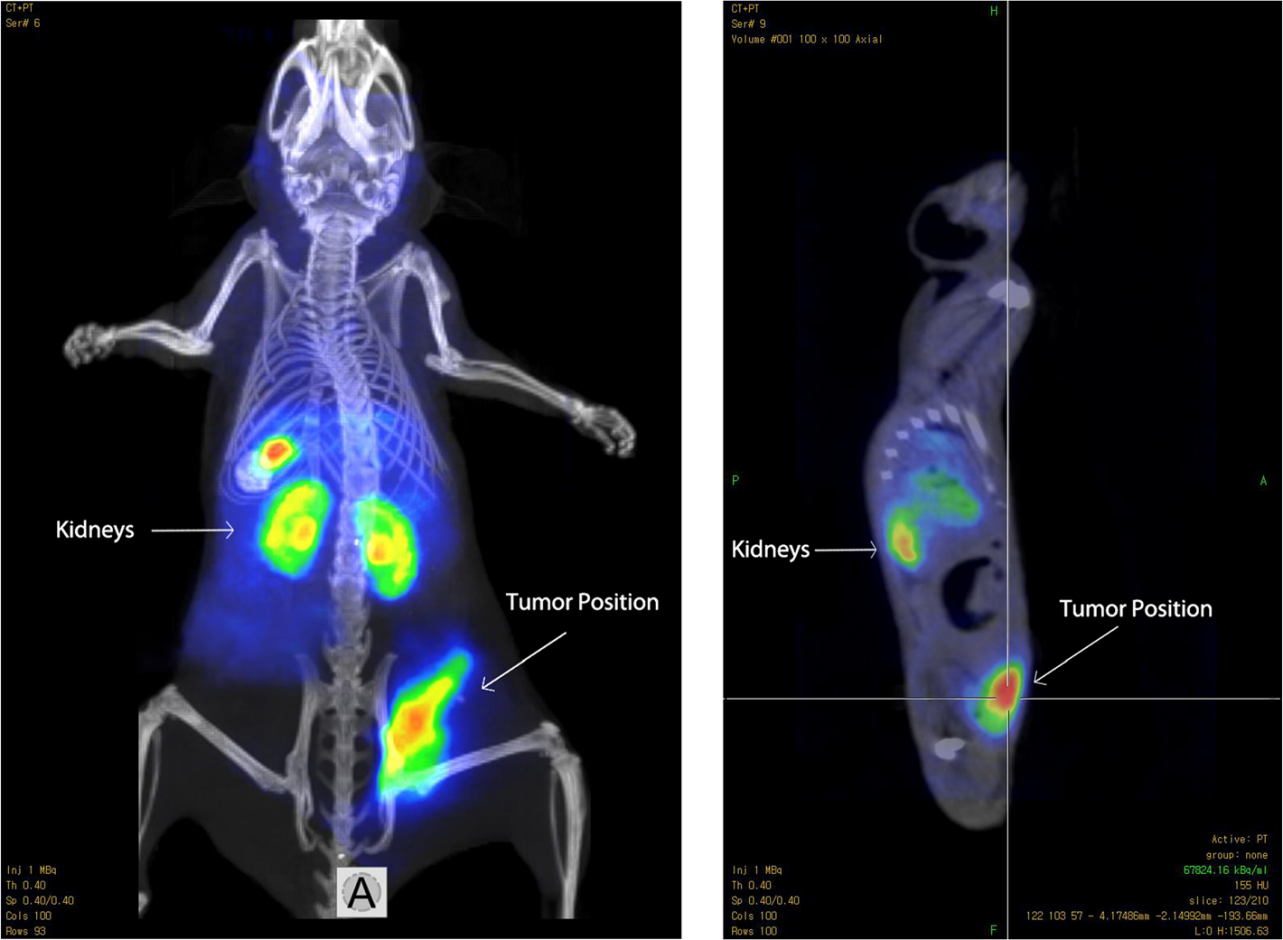
Coronal and sagittal images of tumor-bearing mouse after 1 h Post-injection using 5 MBq MUC1-FA- [^18^F] SFB hybrid peptide conjugate

The initial tumor-targeting efficacy and pharmacokinetic pattern of MUC1-[^18^F]-SFB and MUC1-FA-[^18^F] SFB peptide conjugates were evaluated in SCID mice bearing subcutaneous MCF7 breast cancer cell line xenografts at 1 h with static scans. The uptake of tumor and major organs were quantified based on the analysis of NanoPET/CT images. The tumor uptake image of MUC1-[^18^F]-SFB after 1 h p.i. was visible with a relatively low background. This is attributed to the washout of the radiotracer from adjacent organs and tissue (Fig. 4). Furthermore, the tumor uptake of MUC1-FA-[^18^F] SFB after 1 h p.i. was delineated as compared to the background activity (Fig. 5). These images are concurrent with the finding obtained in quantitative biodistribution data reported in Table 2. The favorable biodistribution profile of MUC1-FA-[^18^F] SFB hybrid peptide conjugate warrants further evaluation and may tempt one to infer that this PET hybrid radiotracer may be useful as a dual receptor-targeting PET imaging molecular probe for breast cancer detection and monitoring tumor response to the treatment.

## Conclusion

We have developed a one-step and rapid synthetic approach for the radiofluorination of the receptor-targeting peptide and their hybrids via carbon atom nucleophilic displacement reactions. MUC1-[^18^F] SFB and MUC1-FA-[^18^F] SFB hybrid peptide conjugates were prepared with radiochemical yields greater than 70% in less than 30 min synthesis time. Radiochemical purities were found to be greater than 95% without HPLC purification, which make this approach amenable for automation and suitable for large scale production. In vitro binding studies on MCF7 breast cancer cell line showed superior affinity of MUC1-FA-[^18^F] SFB hybrid peptide over only MUC1-[^18^F] SFB peptide conjugate. Biodistribution studies in normal mice revealed rapid blood clearance of these radioconjugates with excretion primarily by the urinary system for MUC1-[^18^F] SFB peptide and urinary as well as partially hepatobiliary systems for MUC1-FA-[^18^F] SFB hybrid peptide conjugate. In SCID mice model bearing human breast cancer cell line xenografts, MUC1-FA-[^18^F] SFB hybrid peptide demonstrated excellent tumor uptake and favorable pharmacokinetics over MUC1-[^18^F] SFB peptide conjugate. These observations were confirmed by initial Nano PET/CT imaging with a high accumulation of radioactivity in the tumor. These results demonstrate that MUC1-FA-[^18^F] SFB hybrid peptide conjugate may be useful as a dual receptor-targeting PET imaging probe for breast cancer detection and monitoring tumor response to the treatment, however, further evaluation is warranted.

## Data Availability

All data generated or analyzed during this study are included in this manuscript and its supplementary information files (mass spectrometric analysis and HPLC chromatograms) are also available upon request from the corresponding author.
